# Pain suffering and the self. An active allostatic inference explanation

**DOI:** 10.1093/nc/niae002

**Published:** 2024-02-08

**Authors:** Philip Gerrans

**Affiliations:** Department of Philosophy, University of Adelaide, Adelaide, SA, Australia

**Keywords:** active inference, pain asymbolia, self model, Allostasis, pain disorders, affect

## Abstract

Distributed processing that gives rise to pain experience is anchored by a multidimensional self-model. I show how the phenomenon of pain asymbolia and other atypical pain-related conditions (Insensitivity to Pain, Chronic Pain, ‘Social’ Pain, Insensitivity to Pain, Chronic Pain, ‘Social’ Pain, empathy for pain and suffering) can be explained by this idea. It also explains the patterns of association and dissociation among neural correlates without importing strong modular assumptions. It treats pain processing as a species of allostatic active inference in which the mind co-ordinates its processing resources to optimize basic bodily functioning at different time scales. The self is inferred to be source and target of regulation in this process. The self-modelling account reconciles conflicting deaffectualization and depersonalization accounts of pain asymbolia by showing how depersonalization and pain asymbolia arise at different levels of hierarchical self modelling.

## Introduction

Intuitively pain is an experience of bodily damage that is felt to be unpleasant and aversively motivating. A property of pain that has interested philosophers is its intimate connection with self-awareness. As Wittgenstein said ‘I can only believe that someone else is in pain, but I know [immediately and non-inferentially] if I am’. On this view pain is a condition which is essentially felt to me ‘mine’.

An integrative theory of pain explains how neurocomputational processes give rise to the characteristic phenomenology. Such theories draw on neuroscience, neuropsychology, clinical practice, and the psychology of pain management. Philosophers have recently incorporated this type of evidence to refine philosophical theories of pain. Of particular interest is a disorder called pain asymbolia that raises questions about the relationship between nociception (transduction of sensory signals of damage or threat to bodily integrity), pain sensation, affect, and motivation. Patients with pain asymbolia consequent on damage to the posterior insula cortex say that they are in (often intense) pain but they are not distressed by that pain and they are not motivated to avoid the noxious stimulus. It is therefore an empirical challenge to the intuitive conception of pain. The challenge is increased by reports of pain asymbolia in which subjects report that it feels as if the pain does not belong to them. On some interpretations subjects with pain asymbolia have lost the sense of ‘mineness’ for pain. ‘Mineness’ is a philosophical term of art that refers to the feeling that experiences belong to the subject (Billon 2016, 2023).

In the remainder of this paper I use the related concepts of allostatic active inference and self modelling to develop a neurocomputational account of pain asymbolia. Pain processing turns out to be a particular instance of the general phenomenon of allostatic active inference anchored by a self model. An advantage of this account is its ability to explain the intuitive conception of pain as well as various disorders in which typical features of pain episodes dissociate.

I first describe pain asymbolia and a variety of conditions which pain sensation, distress, aversive motivation, and ‘mineness’ dissociate. I describe how the neuroscience of pain processing accommodates this complexity by treating pain processing in terms of either a modular or a matrix architecture. In the modular conception, pain processing has discrete, dissociable, components responsible for nociception (detection transduction and transmission of signals of threat to bodily integrity) and affective processing. This leads to an elegant ‘deaffectualization’ explanation of pain asymbolia in which affective processing is impaired but nociceptive processing is intact. So the patient still feels the nociceptive aspect of pain but is not distressed by it and consequently not motivated to avoid it. However, dissociations at either the level of neurocircuitry or phenomenology are not as clean as predicted by a ‘strictly’ modular componential processing architecture for pain. And, once we examine a broader range of pain-related phenomena such as chronic pain, congenital insensitivity to pain, distress, and empathy in different conditions, the modular theory of pain processing that underlies the affective theory of pain asymbolia breaks down. So insofar as the deaffectualization theory relies on a modular processing architecture it requires revision.

Problems with a philosophical version of the deaffectualization theory (Grahek 2011) led Colin Klein (2015) to propose a revised account. He proposed that that instead of thinking of asymbolia as a phenomenon of deaffectualization we should think of it as a species of ‘depersonalization for pain’. This fits with reports of depersonalization that resemble reports of asymbolia, like the following.

When a part of my body hurts, I feel so detached from the pain that it feels as if it were somebody else’s pain (Sierra 2009).

I endorse the conceptual revision advocated by Klein’s depersonalization interpretation but it faces related problems. The most persuasive accounts of depersonalization are (i) modular deaffectualization accounts that (ii) explain deaffectualization in terms of hypoactivity in the ‘anterior’ insula cortex, hypothesized to be a neural correlate of distress. But the neural correlate of pain asymbolia is impaired functioning in the ‘posterior’ insula cortex.

I propose a solution that (i) explains the patterns of association and dissociation between pain, motivation, and affect in pain asymbolia and other pain disorders; (ii) explains the relationship between basic bodily (such as nociceptive) and affective experience; and (iii) explains the roles of neural substrates of pain asymbolia (posterior insula) and depersonalization (anterior insula [AI]) and their relationship.

There are two components to the explanation. The first is the idea that pain phenomenology is an emergent result of processing across distributed circuitry baptized the ‘pain matrix’. This matrix is configured in different ways under different conditions, giving rise to the different disorders of pain processing. The idea that pain processing depends on activity in a distributed neuromatrix is fairly standard but its implications for understanding disorders of pain processing are not fully worked out. This paper is a contribution to that project.

The second is the idea that pain processing is a form of allostatic active inference. The concept of allostasis extends the concept of homeostasis to encompass diverse forms of regulatory activity integrated to optimize organismic functioning (Barrett et al. 2016, Corcoran and Hohwy 2017, Kleckner et al. 2017). The concept of active inference is a corollary of allostasis. Active inference treats the mind as [solving] ‘biological regulation problems by learning internal (generative) models of their bodily and interoceptive processes’ (Tschantz et al. 2022, 108266). In the case of pain this means that the brain infers that the self is the thing that senses damage, initiates appropriate internal (regulatory) and external (avoidance) responses, and feels the consequences. To act adaptively in the case of pain many disparate processes ranging from opoid release and cytokine cascade (low-level dynamical processes) to the evaluation of nociceptive signals (an emotional process that gives rise to the distressing aspect of pain) to consulting a Harley St specialist or tiktok influencer must be co-ordinated. The brain solves this problem by attributing signals arising in different modalities to the same unified persisting entity: the self (Moutoussis et al. 2014). This allows a complex system to be regulated as a single entity. When the circuitry that implements this ‘self model’ is identified and its role described, some of the puzzling aspects of pain phenomenology are clarified. This self model is implemented by a pattern of neural activity that integrates bodily emotional/affective and narrative/conceptual processing. Key circuits that sustain this pattern are the posterior insula and AI cortices. The posterior insula is a hub of processing that integrates basic bodily signals including nociception to produce the feeling of embodied selfhood (‘material me’ as Seth (2013) calls it). Activity in the AI links activity in the posterior insula to activity in circuitry that implements emotional and cognitive processes. The resultant interaction between bodily, emotional, and cognitive processing allows ‘raw’ bodily signals to be transcribed into affective signals that inform the subject not just that her body is in a particular state but the significance of that state for ‘her’. For example, an interoceptive signal of bodily arousal can be experienced as exhilaration, anxiety, arousal, or part of an episode of anger depending on the context. I now apply these ideas to explain pain asymbolia.

## Pain asymbolia. Sensation and affect

Pain asymbolia is a rare condition in which patients report a sense of detachment from their experience of pain.

‘In the absence of primary sensory deficits’, these six patients showed a lack of withdrawal behaviours and absence of emotional reactions (or inappropriate emotional responses) to painful stimuli applied to the whole body, as well as to threatening gestures. Five patients also did not react to verbal threats. The ‘patients did not appear concerned about the defect and seemed incapable of learning appropriate escape or protective behaviors’ (Berthier et al. 1988).

The experience is not of insensitivity to bodily damage but rather the feeling that the sensation of bodily damage does not matter to the patient. Sometimes it is described as feeling pain without caring about it or being motivated to avoid it. It has also been compared to feelings of dissociation or anaesthesia. This is consistent with the classic case reported by Schilder and Stengel.

The patient displays a striking behaviour in the presence of pain. She reacts either not at all or insufficiently to being pricked, struck with hard objects, and pinched. She never pulls her arm back energetically or with strength. She never turns the torso away or withdraws with the body as a whole. She never attempts to avoid the investigator (Schilder and Stengel 1931).

Classic neuropsychological accounts (sensorimotor-limbic disconnection accounts as they are known) treated pain asymbolia as evidence for the idea that pain processing has a modular architecture with a ‘sensory’ and an ‘affective’ component (Mesulam 2000). This wave of theorizing about pain asymbolia is anchored in neuroscientific explanation of pain, particularly conditions like chronic pain, placebo analgesia, congenital insensitivity for pain, and empathy for pain. Each of these phenomena has been elegantly explained on the assumption that ‘sensory’ pain and ‘affective’ or ‘emotional’ pain are produced by distinct components of a specialized pain processing system. On one version of this modular view, pain asymbolia is the result of loss of affective response to noxious stimuli. This would explain why asymbolic subjects do not react aversively to pain or experience it as distressing, even though they continue to sense damage to their body.

The syndrome of asymbolia to pain appears to be a somatosensory-limbic disconnection syndrome. Pain asymbolia can be conceptualized as a somatosensory analogue of visual ‘hypoemotionality’ (Sierra et al. 2002).

This modular explanation of pain processing and pain asymbolia accommodates a consensus about its neural substrates. Nociceptive input is processed via distinct (but not discrete) neuroanatomical pathways. A sensorimotor pathway conveys nociceptive information to the somatosensory cortex and insula. Another projects to the insula, the paralimbic and limbic systems, and ultimately prefrontal cortex. At the same time, activity in these pathways can be influenced by descending modulatory systems that target different aspects of the system at different time scales. A circuit linking the AI and rostral anterior cingulate cortex plays a crucial role in sustaining the feeling of distress and linking it to higher level (narrative and conceptual) forms of self-awareness. Thus, there is preliminary evidence that the distress (‘souffrance’) of pain and the sensation of bodily damage (‘douleur’) are potentially dissociable because their substrates, although they communicate in the construction of the pain experience, are distinct (Berthier et al. 1988, Danziger 2006, Starr et al. 2009, Klein 2015, Gerrans 2020).

## Evidence for dissociations

In support of the idea of distinct and dissociable pathways for sensorimotor and affective processing, the modular theorist can point to conditions such as chronic pain. Chronic pain is explained as a form of neuroplastic change in which circuitry involved in affective processing ‘takes over’. Such cases are described as ‘nociplastic’ rather than nociceptive.

According to a recent meta-analysis, the experimental induction of acute pain (e.g. with painful versus non-painful thermal stimuli) is generally associated with activations of both sensory (e.g. thalamus, secondary somatosensory cortices (SII), dorsal posterior insula) and affective (e.g. dorsal anterior cingulate cortex [ACC], AI) brain regions in healthy adults and pain patients (Xu 2020).

In contrast, nociplastic pain conditions, such as nonspecific chronic low back pain (cLBP), are associated with altered neural activation patterns in ‘affective brain regions only’, particularly the rostral ACC, mPFC, and amygdala (Gu et al. 2013).

This is consistent with the classic modular conception in which the primary substrate for suffering is a circuit centred on the AI and anterior cingulate cortices. Indeed, a last ditch treatment for chronic pain is lobotomy that disconnects this circuit from prefrontal regions. Patients treated by lobotomy reported that the intensity and nature of the pain had not changed and their automatic responses to a noxious stimulus were ‘intact and often exaggerated’. However, they are untroubled by pain or the prospect thereof. As Danziger (2006) puts it ‘[E]motional impact of chronic pain is dramatically reduced’.

Cases such as this form part of

a wealth of evidence suggesting that patients with chronic pain may have anatomical alterations within regions involved in cognitive and emotional modulation of pain, such as the dorsolateral and medial PFC, the ACC, and the insula (Barrett 2017).

Another consideration that supports this is the action of morphine analgesia. In low doses, the primary target of morphine is

activations in the amygdala, AI, and cingulate cortices, ‘which are regions implicated in the affective aspects of pain’, are maximally suppressed at the lowest opioid dose (Lee et al. 2014, Rütgen et al. 2015).

This is consistent with the idea that the AI-ACC circuit is implicated in personal distress. This form of suffering can be produced and regulated relatively independently of low-level nociception and reflexive behavioural responses like flinching and withdrawal.

These studies suggest that pain processing occurs along different dimensions with sensorimotor responses occurring earlier and outside the scope of deliberate control. A primary hub of this processing is the ‘posterior insula’, consistent with its role in integrating low-level bodily signals crucial in maintaining organismic viability. Threats to bodily integrity detected in nociception are processed by the posterior insula since they are vital to homeostatic regulation (Gu et al. 2013, Frot et al. 2014). It is crucial to emphasize this point because the posterior insula is ‘not’ hypothesized to be the substrate of the experience of distress but of an earlier stage of pain processing that integrates the nociceptive signal with other bodily signals as part of a system of basic bodily regulation.

The feelings of suffering and distress that are part of the pain experience are produced at a higher level or later stage of processing that involves the AI, modulated by prefrontal cortical structures that represent personal and social information and explicit narrative and conceptual self-knowledge. If this is correct, then one can see the appeal of the modular account. On the assumption that the mechanisms that support these distinct aspects of processing are relatively specialized and can be selectively activated or damaged, one can see why it makes sense to partition pain processing into sensori-motor and affective-personal components. However, such an account needs to explain why pain asymbolia is associated with damage to the posterior rather than AI. After all, on the modular account, it is the activity in an AI that produces feelings of distress. So one might predict that pain asymbolia would be the result of damage/dysfunction in anterior rather than posterior insular regions. I return to this question below.

## Empathy and chronic pain

The modular deaffectualization account can point to further evidence that the AI is the substrate of distressing experience. Some of the most dramatic evidence comes from studies of patients who are congenitally insensitive to pain and patients with congenital agenesis of body parts. Such patients clearly lack early sensory processing of bodily damage, either because they lack activity in the necessary sensorimotor processing circuitry or lack the body part itself. Nonetheless, when viewing images of bodily damage, the patients experience distress. This can be described as a form of empathy where empathy is taken to mean the ability to experience affective states while observing, but not actually experiencing, sensory elicitation of those states. i.e. seeing or imaging someone else (or oneself) in pain or distress.

Viewing body parts in pain corresponding to the missing limb induces a significant activation ‘only in brain regions devoted to emotional empathy, such as the anterior insula’ (Betti and Aglioti 2016).

Interestingly, however, in the absence of a somatic representation, i.e. perception of the body part, ‘the understanding of another’s pain relies on inferential mechanisms rather than affective resonance mapping’ (Betti and Aglioti 2016, 195). The idea behind this enigmatic sentence is that empathetic distress in the absence of nociception can be driven by higher level cognition, for example by thinking about others’ painful experiences.

The authors of this review conclude that both perception of other’s pain and experience of pain are typically initially processed by sensorimotor structures and subsequently processed by an affective system that evaluates the significance of bodily damage for the subject. However, in the absence of initiating sensorimotor processing, the affective system remains able to produce a feeling of distress for a perceived or inferred injury ‘to self or other’.

Tania Singer and collaborators asked female members of couples to observe their partners experiencing a mild electrical shock to the wrist. They were not explicitly instructed to empathize, only to observe. Patterns of neural activation were compared to a condition in which they received a shock themselves. The key difference reported was that

only part of the network mediating pain experience is shared when empathizing with pain in others. Empathizing with someone else’s pain elicited activity principally in left and right AI, ACC, lateral cerebellum, and brainstem (Singer et al. 2004).

The main contrast with the ‘self’ pain condition was that

pain-related activation in contralateral SI, SII/posterior insula, and caudal ACC are specific to self-experienced pain, as opposed to perceived pain in others.

Their conclusion is a very clear statement of the view that pain processing has dissociable sensorimotor/discriminative and affective components and it is the latter that are involved in empathy.

(Zhou et al. 2020) Rostral ACC and AI appear to reflect the emotional experience that evokes our re-actions to pain and constitutes the neural basis for our understanding of the feelings of others and ourselves. (Singer et al. 2004 1161, my italics).

that connectivity between mPFC and regions of the salience network (SN), including the insula and ACC, were found to be increased among patients with nociplastic pain compared to controls (Yarns et al. 2022 104558).

(Wang et al. 2020) The idea that an ACC-AI circuit is the substrate for feelings of distress is also part of the explanation of chronic pain (Simons 2014). For example, a meta-analysis of studies comparing patients with and without chronic pain reported a consistent role of the AI consistent with its ‘complex role in processes directly or indirectly related to the acute and chronic pain experience, including pain empathy (Fallon et al. 2020; Xu 2020; Zhou et al. 2020), interoception and salience processing (Li et al. 2020; Yao et al. 2018) as well as emotional experience (Gogolla 2017; Ferraro et al. 2022). Another study of emotional regulation and pain processing found (Duquette et al. 2007; Yao et al. 2018).

As a consequence, the most effective interventions for chronic pain do not target nociceptive processing pathways or posterior insula where they converge and are integrated with other basic forms of bodily signalling. Rather, the most effective treatments are aimed at processes of emotional regulation and reappraisal that modulate activity in the AI-ACC.

Thus, a modular view of pain processing can point to evidence of selective activation and independent regulation of sensorimotor and affective components of a pain processing system. The substrates of these systems are circuits involving posterior insula and AI, respectively. On this view, Pain Asymbolia and Chronic Neuroplastic Pain represent a form of double dissociation. Asymbolia is evidence of preserved sensory processing and absent affective processing, and Chronic Neuroplastic Pain is evidence of reduced sensory processing and intact or exaggerated affective processing.

Affective states are typically thought of as ‘intrinsically’ motivational. We avoid distressing situations and approach pleasant ones. This would explain why patients with pain asymbolia, who are not distressed by pain sensations, lack aversive motivation. However, as Klein (2015) among others points out, if affect is intrinsically motivating, lobotomized patients who have flattened affective responses should lose their aversive reaction to noxious stimuli. However, their reflexive aversive responses are intact or exaggerated (Danziger 2006; Duquette et al. 2007). So, ‘there is no necessary connection between affective experience and aversive behaviour’.

Similarly, patients with chronic insensitivity to pain have intact affective circuitry but they have no motivation to avoid painful stimuli, typically with disastrous results. ‘*She* reported *numerous burns and cuts without pain (Supplementary Fig. S1), often smelling her burning flesh before noticing any injury*’, Br J Anaesth. 2019 Aug; 123(2): e249–e253.

## Against the modular theory of pain asymbolia

Cases like these suggest that processing of the nociceptive signal represents the brain’s response to the significance of the stimulus in that context, with sensory, affective, behavioural, and cognitive processes all contributing. However, in non-standard contexts, elements of this processing system can dissociate in different ways. But this does not license the modular hypothesis of discrete dissociable components of a specialized pain processing system. When we turn to a wider range of evidence about pain processing, we see ‘interdependence rather than independence’ of sensorimotor and affective aspects of pain processing.

One illustration of this coupling is provided by empathetic response in different conditions. Recall that Singer’s experiments used couples observing a partner receiving a low-intensity painful stimulus. In that condition, the circuitry activated by both observation and personal experience was the AI/rostral ACC, the hypothesized basis of distress. However, in other conditions in which subjects view severe injuries or wounded body parts, somatosensory aspects of the pain processing system are also activated (Betti and Aglioti 2016). This is consistent with some somatosensory resonance or contagion conceptions of empathy that emphasize that third person observation and first person experience of body state can activate the same sensory processing systems (Singer and Lamm 2009).

The case of ‘social pain’ evoked by ostracism or criticism makes a similar point. It is natural to conceive of it in terms of affective processing: the distress evoked by rejection. As such, on a strictly modular view, one might predict activity in the AI-ACC associated with affective experience, and this is confirmed. However, social pain can also activate the sensorimotor system, suggesting that ‘high level personal representations can entrain sensory processing in the absence of eliciting noxious stimulus’ (Eisenberger 2012). These cases suggest a complex, continuous structure to pain processing whose elements can be co-ordinated in a context-sensitive way.

We can add to this that most of the processing of pain signals is not performed by circuits specialized for responding to noxious stimuli. Aversive responses to pain are sensori and viscero-motor. Attentional and inhibitory processes are amodal. For example, dorsolateral prefrontal structures have an essential role to play in pain modulation shown by the fact inhibition of activity in these circuits removes the placebo response. These structures do not work in isolation but in co-operation with ventrolateral and orbitofrontal structures involved in cognitive reappraisal and modulation of affective response and sensorimotor responses. ‘The descending modulatory systems involve brain regions that are important not only for pain but also for cognitive and emotional functioning in general’(Bushnell et al. 2013). In other words, the regulatory role for dorsolateral circuitry is not restricted to pain modulation.

Similarly, empathetic responses, both affective and sensorimotor, depend on activity in systems that one might think of as, strictly speaking, not dedicated to processing noxious stimuli. Empathy is strongly modulated by attachment and by the interpretation of others mental states and attitudes, which in themselves have nothing to do with nociception. This is why the science of pain has moved away from explaining pain experience as the result of processing in a system specialized for pain processing with discrete sensory and affective components.

## The pain matrix

The concept of a pain matrix was introduced by Melzack (1990) in the context of explaining the persistence of phantom limb pain and its resistance to anaesthesia. He explained the persistence of painful experience in the absence of body parts in terms of a distributed ‘neuromatrix’ that extends ‘through selective areas of the whole brain including the somatic, visual and limbic system’. The activation of elements of the matrix in the absence of sensory input can lead to exaggerated pain experience, not least because there is no possibility of relieving a precipitating bodily injury through movement or anaesthesia. Melzack argued that the structure of the matrix is partly genetically specified (the ‘pain connectome’) but its final architecture is sensitive to developmental influences. The result is that the matrix can be activated via a variety of pathways depending on idiosyncratic patterns of connectivity and conductivity. Chronic pain and ‘social’ pain for example are evoked by different sources and sustained by a different pattern of activity across the matrix to pain directly evoked by injury. The main point is that different types of pain experience should be thought of as resulting from different ‘patterns of activity across the whole network’ rather than as evidence of a modular architecture for pain (Melzack 1990). In particular, a strict double dissociation between sensorimotor and affective processing is not supported by the evidence.

The matrix conception also undermines a strict bottom up or feedforward model of pain processing in favour of one in which pain experience is the emergent produce of recurrent processing across the matrix. ‘Social’ pain occasioned by ostracism or criticism is an example. It is typically associated with activity in the AI circuitry which suggests an ‘affective’ interpretation: the subject is feeling personal distress. However, there are also cases in which social pain activates posterior systems involved in sensory processing (Eisenberger 2012). One explanation is

the role of the dACC and AI in responding to socially painful experience; these regions may be crucial for ‘translating’ experiences of social disconnection into downstream physiological responses, which have implications for health. Indeed, the dACC and AI may have a mediating role in the links between social rejection and both inflammatory activity and depression (Eisenberger 2012).

This interpretation treats the AI as an integrative hub that functions as a relay station between bodily regulation, emotional appraisal, and conceptual and narrative forms of self representation. Eisenberger is suggesting that this intermediary role for the AI goes both ways. Not only does the AI transcribe interoceptive and nociceptive experience to integrate bodily regulation adaptively with higher level processing, it also transcribes high-level personal and social and personal information (for example about ostracism or rejection) into formats that allow for adaptive low-level bodily responses. On one way of reading Eisenberger, the nociceptive signal can be processed bottom up from sensorimotor to conceptual-social levels or top down from conceptual-social to sensorimotor. In both cases, the AI is an intermediate hub or relay station.

The matrix conception suggests that pain processing activates distributed circuitry across an essentially amodal matrix whose elements include hubs of bodily/interoceptive, social emotional, and conceptual and executive processing. Activity across this matrix can be driven from any starting point. Anxious rumination for example can make people prey to a range of distressing bodily and emotional experiences, including the amplification of innocuous nociceptive signals (Terasawa et al. 2013). Or, in prototypical cases of pain, it can be driven by perception noxious stimuli such as a burn or broken bone. In either case, the ultimate experience ‘reflects the pattern of activity across the matrix’.

Nociceptive cortical processing is initiated in parallel in sensory, motor, and limbic areas; it progresses rapidly to the recruitment of AI and fronto-parietal networks, and finally to the activation of perigenual, posterior cingulate and hippocampal structures. Functional connectivity between sensory and high-level networks increases during the first second post-stimulus, which may be determinant for access to consciousness (Garcia-Larrea and Bastuji 2018).

The fact that there is a typical sequential pattern across the matrix from peripheral (nociceptive) to more central processes encourages the idea of an hierarchical modular structure to pain processing if we concentrate on standard cases and neuropsychological deficits. However, the matrix explains these cases in terms of the role of hubs of cortical and subcortical processing whose activity can be initiated and maintained in different ways in different contexts. The posterior insula is an integrative hub for basic bodily regulation of which nociceptive signals form an important class. The role of the AI here is as a crucial integrative relay station that allows bodily changes to be evaluated for personal significance and social/personal information to entrain appropriate bodily responses. In order to play that role, the AI communicates with hubs of circuits such as amygdala and ventromedial prefrontal cortex that orchestrate emotional appraisal. As a recent review put it the insula is not an isolated ‘island’ but rather an integral brain hub connecting different functional systems underlying sensory, emotional, motivational and cognitive processing (Gogolla 2017 585).

Activity across the insula reflect different ‘levels’ of integration with posterior insula a convergence zone for basic bodily signals and the AI a relay station between posterior insula and systems that determine the significance of those signals personal/social goals. Partitioning of the insula is not sharp but continuous.

The activation associated with both pain-related (posterior insula) activation and that associated with prediction error (PE)-related (AI) activation correspond well with connectivity gradients observed along the posterior–anterior axis (Horing et al. 2022).

This matrix conception also helps explain how it is that the Default Mode Network (DMN) can play a crucial role in modulating the nociceptive signal. The DMN is a hub of autobiographical representation and crucial substrate of the narrative self (Davey et al. 2016). As such, within a broadly modular framework, one might regard its functioning as independent of sensory processing. But, in fact, it plays an important role in pain regulation largely through its interactions with attentional and modulatory systems.

Activation in the salience network was found when attention spontaneously focused on pain (20). In contrast, the DMN was engaged when attention was focused away from pain (20). ‘Individuals’ intrinsic attention to pain’ (defined by the test–retest reproducibility of an individual’s tendency to attend away from pain) was related to their structural and functional connectivity between DMN and the descending pain control system (and the PAG in particular) (18). Also, alterations in the interplay between the salience network, DMN, and descending pain control network have been related to heightened attention to pain in chronic pain patients (Wiech and Tracey 2013).

## The ‘pain’ matrix and the role of self modelling

When Melzack introduced the idea of a matrix, he treated it as the basis of a bodily sense of self. On his view, the matrix explains the fact that

experience of the body has a unitary, integrated quality that includes the quality of the ‘self’, the feeling that all the parts of the body are uniquely one’s own (Melzack 1990).

Melzack was explaining why a person with a phantom limb still experiences that limb ‘as hers’. In order to act to avoid or reduce damage, the brain needs to treat experience produced by the matrix as a property of a unified persisting entity: the source of bodily experience and target of regulation (Metzinger 2003; Gerrans 2014; Menon and Uddin 201; Limanowski and Blankenburg 2013; Hohwy and Michael 2017; Seth and Tsakiris 2018). In the case of phantom limb, the model fails to update after amputation.

The matrix idea fits with the view that the basic processing properties of the brain depend on the synchronized activity of large-scale networks (Pessoa 2017). These networks comprise (at least) the salience network (allocation of cognitive resources whose top level is the AI-ACC circuit) the default mode network (explicit episodic self-referential processing) and an executive network (high-level cognitive control) (Menon and Uddin 2010; Gerrans 2014). Each of these networks has a proprietary network architecture (Betti and Aglioti 2016). When we apply this idea to the processing of pain, the idea that there is a ‘sensorimotor circuit’ for initial processing of processing noxious stimuli and a discrete ‘affective circuit’ centred on the AI-ACC dissolves. Instead, the insula functions as a relay station between the salience system, sensorimotor processing, and the default mode network to help configure activity in the matrix. This is consistent with the polymodal involvement of the insula in

response selection, interoception, attention, response conflict, autonomic arousal. Tellingly, functions non-specifically linked to pain are also carried out by the AI, which is implicated in cognitive, affective, and regulatory functions, including interoceptive awareness, affective response, empathic processes, and uncertainty (Betti and Aglioti 2016, 198).

Activity in the AI provides a constant ‘locus of concern’ ensuring that activity across the brain is integrated for the organism’s benefit. This does not mean that the AI is a ‘self module’, just that the feeling of being a unified self is an emergent property of the integration of distributed activity to serve the goals of the organism and that integration represents the system as a unified entity. This view is more consistent with the allostatic active inference view of cognition which treats the mind as actively engaged in finding a pattern of neural activity that optimizes functioning of the organism. The self is inferred by the mind to be ‘the entity who benefits’ when predictions are realized. When the AI is not active, sensation and cognition are not impaired but activity across the matrix is not integrated to serve the goals of the subject. The resultant experience is reported with a sense of detachment or depersonalization. Allostatic active inference has lost its centre of gravity.

## Asymbolia as depersonalization

This framework supports the argument of Colin Klein against deaffectualization theories of pain asymbolia. He suggested that asymbolia is a species of ‘depersonalization for pain’. As he says

They recognise it as pain, but in some important sense it has ceased to be something worth caring about. It thus has the feel of a sensation *which they can no longer identify with as their own* (Klein 2015).

In this respect, his account recalls the explanation of classic depersonalization (DPD) experience by (Michal et al. 2014) as ‘difficulties of DPD patients to integrate their visceral and bodily perceptions *into a sense of their selves*.’ In DPD, the inability to model experience as belonging to the self is global so that patients will report feeling detached from ‘all’ experiences including pain. In pain asymbolia that detachment is reported as applying specifically to pain. So the ‘bodily perception’ not integrated into a sense of self is the nociceptive signal. This is why Klein (2015) argues that asymbolia is a form of depersonalization restricted to pain. As he puts it:

the phenomenology of asymbolia might resemble a kind of depersonalization syndrome. … The asymbolic, and the depersonalized more generally, feel sensations that they are estranged from—that they do not take to be theirs in the sense that we normally do. … (Klein 2015).

This view, while attractive, is initially hard to square with neuropsychiatric theories of depersonalization that treat it as a form of ‘dissociative deaffectualization’. For these theories, depersonalization results from involuntary inhibition of the AI as a dissociative response to intractable adversity such as trauma or abuse (Sierra 2008, 2009, Medford and Critchley 2010, Medford et al. 2016). In other words, depersonalization theorists tend to endorse a version of the modular theory of affective processing and treat depersonalization as a loss of affect resulting from hypoactivity in the AI. This strategy exploits the brain’s natural opioid system (Sierra 2008). Morphine analgesia has the same dissociative effect and seems to initially exploit the brain’s distress regulation systems (Lee et al. 2014, Rütgen et al. 2015)

by initially targeting the AI. The functional magnestic resonance imaging (FMRI) data suggest that opioid analgesics can directly influence emotional responses at low doses that do not alter sensory aspects of pain (Lee et al. 2014).

In general, depersonalized patients who are deaffectualized have intact basic bodily awareness and regulation. A reasonable inference is that their posterior insula functioning is unimpaired but they feel depersonalized in virtue of hypoactivity in the AI. That is the deaffectualization theory of depersonalization in a nutshell. Recall however that Klein proposed the depersonalization theory of asymbolia as an ‘alternative’ to the idea that it is a form of deaffectualization for pain. We should also recall that asymbolia is agreed on all sides to be the result of damage to posterior insula, hypothesized to be an integrative hub for basic bodily regulation not affected.

## Reconciliation. The self model

These facts are hard to reconcile *prima facie*. However, the role of the insula in self modelling explains them. The posterior insula cortex integrates values of basic bodily variables like blood pressure and hydration as well as nociception (bodily damage) to co-ordinate basic regulatory functions. The posterior insula is thus the primary substrate of interoceptive experience and hence of what Anil Seth called ‘material me’, the feeling of being an embodied self. The AI cortex integrates interoceptive signals from the posterior insula with information from other channels. This enables bodily signals (such as nociception) to be contextualized and managed adaptively by entraining the full range of regulatory capacities. The result is that we do not just feel like the subject of bodily states but of emotional states that reflect our goals. In a dangerous episode, we do not just feel adaptive bodily responses but we feel them as fearful because those bodily responses are produced as ways of realizing a goal of avoiding danger.This is why the concept of allostatic active inference helps explain why the pain matrix, though essentially amodal, can give rise to such various phenomenology. Allostatic active inference is a process of recruiting and co-ordinating a suite of systems to maintain systemic integrity. In the case of pain this means detecting, avoiding, responding to, and repairing damage using relevant resources. This requires integrating relevant systems. At the most basic level, managed by the posterior insula, this integration is felt as intero/nociceptive experience and embodied selfhood. The AI relays the interoceptive signal to other systems that help interpret and manage it adaptively. This transcribes the interoceptive signal allowing it to be experienced as an affect. For example, an abdominal cramp will be felt quite differently if it is experienced transiently in the gym doing sit-ups or if it is experienced by someone who is fearing a miscarriage.

Thus, it is not surprising to see that contemporary affective neuroscience treats experience produced by activation of the AI as a form of higher order bodily representation that represents the integrated functioning of the organism ‘evaluated against emotionally salient goals creating a sense of self in the process’. As Bud Craig (2009) puts it,

the integration successively includes homeostatic, environmental, hedonic, motivational, social, and cognitive activity to produce a ‘global emotional moment’, ‘which represents the sentient self at one moment of time’ (Craig 2009).

One difference between Craig’s account and mine is that Craig’s account almost treats the AI as a discrete ‘self module’. My view is the more modest one that the AI is an integrative hub whose activity transcribes the interoceptive signal into one that signals emotional salience. The emergent result of this integrative process is the experience of being the subject of salient experience.

To return to the case of pain asymbolia, in pain asymbolia, dysfunction of posterior insula leads to failure to model nociceptive signals as belonging to the bodily self. This produces the experience of sensations of bodily damage (or other forms of threat to bodily integrity) that do not belong to ‘me’. This failure to incorporate nociceptive signals at the level of bodily self-modelling means that they do not entrain regulatory responses, including higher levels of active inference that depend on AI activity. If the signals are not sensed as belonging to me at the most basic level of selfhood, there is no need to establish their emotional salience. Consequently, they are not affectively transcribed. So, they do not lead to the feelings of personal distress and attempts to reduce them. The narrative I report is the sensation of nociceptive experience that is not felt to matter to the self. Pain aysmbolia is thus a case of processing nociceptive signals ‘outside’ the self-modelling hierarchy. That is to say without those signals being attributed to the self whose goal structure determines the regulatory response to sensory experience. This explains why, as Klein (2015) puts it, asymbolia is a kind of ‘[I]ndifference. One’s body becomes, as it were, just another object in the world’.

## Data Availability

No new data were generated or analysed in support of this research.
